# Rates of IUCD discontinuation and its associated factors among the clients of a social franchising network in Pakistan

**DOI:** 10.1186/1472-6874-12-8

**Published:** 2012-03-29

**Authors:** Syed Khurram Azmat, Babar Tasneem Shaikh, Waqas Hameed, Mohsina Bilgrami, Ghulam Mustafa, Muhammad Ali, Muhammad Ishaque, Wajahat Hussain, Aftab Ahmed

**Affiliations:** 1 Marie Stopes Society, karachi, Pakistan; 2 Marie Stopes Society, 21-C, Commercial Area, Old Sunset Boulevard, Phase II, Defense Housing Authority, 75500 Karachi, Pakistan

**Keywords:** Intra-uterine contraceptive device, Clients' satisfaction, Contraception, Family planning counseling, Social franchising

## Abstract

**Background:**

Modern Intrauterine contraceptive device (IUCD) is very safe, highly effective reversible and inexpensive family planning method which offers 5-10 years of protection against pregnancy. The contraceptive use in Pakistan has been merely 30% for over a decade with IUCD being the least used method. Higher discontinuation rates are documented in developing countries; however no such data is available for Pakistan. Marie Stopes Society (MSS) established a social franchise outlets network branded as '*SURAJ' *(Sun) in Pakistan to provide quality family planning services. This study attempts to determine IUCD discontinuation rates and its associated risk factors. Using a semi-structured questionnaire, a cross-sectional study was conducted with 3000 clients who availed IUCD services from *Suraj *provider 6, 12 and 24 month back,. Data were analyzed in SPSS 17.0; adjusted prevalence ratios were calculated to see associations between discontinuation and its risk factors.

**Case presentation:**

We found that 22.7% of the IUCD acceptors experienced some health problem; while the overall discontinuation rate was 18.9% with average time of usage of 7.4 (SD ± 5.8) months before discontinuation. Half of them showed health concerns (49.8%); of which a majority (70.2%) returned to *Suraj *provider for IUCD removal. Women living in Punjab, residing at a travelling time of 30-60 minutes and no previous use of contraceptive are more likely to discontinue IUCD. However, among total women 81.7% still expressed willingness to avail IUCD services from *Suraj *provider in future, if needed.

**Conclusion:**

The findings suggest a need for training the providers and field workers to prevent early discontinuation of IUCD among the *Suraj *clients and by addressing the health concerns through proper counseling, continued follow-up and immediate medical aid/referral in case of complications.

## Background

Modern Intrauterine device (IUCD) is very safe, highly effective (99%) during first year [1] and inexpensive family planning method [2]. It is reversible, requires little effort on the part of the user once inserted, and offers 5-10 years of protection against pregnancy. IUCD's wider use would reduce the overall number of unintended pregnancies more than any other method. Globally, after sterilization (19%), approximately 13% of all women of reproductive age use the IUCD, making it the second most popular contraceptive [3].

However, it has a bad reputation in some countries with its adverse effects exaggerated and the advantages often under-stated [4]. Fears about side effects, concerns about infection and infertility, lack of technical training for providers, and the time and costs involved in providing services combine to discourage use of IUCDs in many countries [5]. Some studies revealed that up to 80 percent of IUCD users complain of increased menstrual bleeding and pain. However, research conducted by FHI in 23 developing countries, indicates that Copper T IUCD-related bleeding disturbances tend to decrease after the first few months of use [6]. On the whole, researches around the world indicate the difficulty of IUCD use, continuation, within first year removal [7,8]. Introducing or reintroducing a contraceptive requires attention to policy, counseling and service delivery, on one hand, and to the public and potential users' knowledge and perceptions, on the other. A comprehensive approach that brings well informed clients together with good-quality services will help ensure a successful IUCD program [3].

### The context

Pakistan is one of the most densely populated countries with a population exceeding 180 million with majority of the population (65%) living in rural areas [9]. Moreover, Pakistan's population growth at 1.9% per annum is much higher than that of its South Asian neighbors. It currently has the largest cohort of young people in its history (25 million, aged 15-24) and subsequent cohorts are projected to be even larger. Moreover, more than half of the women in Pakistan enter into marriage before reaching age 20 and one-third marry before they are 18 years of age [10]. However, the country lags far behind on development indicators, particularly with regard to maternal and child health [11]. Seeking health care from a quality services outlet would mean an extra financial burden, resulting in inequities in access to care and low utilization of needed services and products [12].

Against almost universal contraceptive awareness, overall contraceptive prevalence is reported to be at 29.6%; while, the use of modern contraceptive methods is reported to be even lower at 21.7%. Moreover, almost half of currently married women have used contraceptives (modern or traditional methods) at one time, indicating that a significant share of women have discontinued use of family planning. The higher total fertility rate at 4.1 as against 3.1 wanted total fertility rate indicates that on average women in Pakistan bear at least one unwanted pregnancy in their lifetimes [10]. In 2001, after male condoms, IUCDs were the most popular reversible modern contraceptive method in Pakistan, representing 17% of the method mix, compared with 11% in the mid-1980s [13]. However, according to a national survey in 2003, the awareness pertaining to IUCD is quite low at 74.8% compare to overall contraception knowledge, and interestingly, IUCD is the only method whose awareness among population has reduced. Likewise, the use of IUCD has also reduced from 4% in 2000 to 2.3% [14].

The most common contraceptive methods in use in Pakistan are either permanent or have low effectiveness, these include: female sterilization (8.1%), traditional methods such as rhythm and withdrawal (8%), and condoms (7%). Contraceptive use is even lower among young and rural women. Twenty five percent of the people living with unmet need for contraception; meeting this demand can have immediate effect reducing Pakistan's total fertility rate (TFR) to three births per woman thereby putting it on the trajectory for reaching replacement fertility in the near future. Moreover, the median birth interval is 29 months and that can potentially be addressed through the long term reversible method in Pakistan [10].

### Rationale for the study

Under the auspices of Marie Stopes Society (MSS) a health providers' network branded as '*Suraj*' (meaning 'sun' in English), with an innovative Output Based Aid (OBA) voucher scheme for IUCD services has been established. The model is essentially a partnership between MSS and private local health services providers and aims to increase demand, access, choices and quality services of family planning for rural, underserved and poor communities, similar to a "social franchise model". MSS *Suraj *network is operating in total eighteen 18 districts (6 each; in southern & northern Punjab, and Sindh). In each district' the network ranged from 4-7 providers those are located in the far-flung, rural and underserved areas of the district, and each provider covers a total of 15,000 to 25,000 population. In each district the minimum distance between any two *Suraj *providers is large enough to ensure no spill-over effect. The Suraj network forged partnerships with 100 Private providers which mainly included Lady Health Visitors. The providers belonged to and were practicing in rural, poor and underserved areas. *Suraj *providers were trained and accredited to provide condoms, emergency contraceptives, injectables, oral contraceptives and to insert and remove intrauterine contraceptive devices (IUCDs). Since its inception the *Suraj *network has provided nearly 106,943 IUCDs in its catchment area including 30% through vouchers and 45% are to the women, referred by field worker mobilization (FWM). Thus, in order to identify strategies for augmenting the efficiency and effectiveness of this program, this study was an attempt to assess the frequency of discontinuation of IUCDs, its reasons. Moreover, clients' level of satisfaction, and accessibility to the services were also assessed.

### Objectives of the study

The main objective of this study was to assess the outcomes of IUCD use and to determine the factors associated with IUCD discontinuation among women clients of *Suraj *clinics.

### Methodology

#### Study design & setting

A cross-sectional study was conducted in nine districts of Sindh and Punjab Provinces (6 from Punjab & 3 from Sindh). The districts from Punjab province included Bahalwanagar, Jhang, Kasur, Lodhran, Sheikhupura, and Sialkot; while from Sindh, Umerkot, Hala/Matiari and Tando Muhammad Khan were included in this survey. Geographically, Bahawalnagar, Jhang and Lodhran are situated towards the southern part of Punjab province whereas Kasur, Sheikhupura and Sialkot are more towards the northern region of the province. In each district, a sample of *Suraj *providers was selected for this survey.

#### Sampling

A multi-stage sampling procedure was employed. At first, three districts were randomly selected proportionally from each region (southern & northern Punjab, and Sindh). Within each district, we selected *Suraj *centres that had (averagely) performed at least 25 (monthly targets) IUCDs in three selected cohort (i.e. 6 month, 12 month and 24 month back). The purpose for choosing 6 month was to compare the findings with an outreach evaluation conducted by MSS earlier [15], while 12 and 24 month discontinuation rates were to compare with other developing countries [16-18]. The daily record registers of each selected provider for these specific cohorts were obtained from the field and entered on an Excel sheet (version 2007). Finally taking into account the operational and financial factors, from a list frame of women (who received IUCD), a total sample of 3000 clients were randomly selected after stratification by type of client; which represent 55% of voucher clients and 45% clients referred by field worker marketing. Since the model itself was innovative and MSS employed the voucher distribution for the first time; therefore the purpose to keeping higher share of voucher clients in selected sample was to validate the distribution of voucher.

#### Inclusion criteria

Women of reproductive age (15-49 years) who had received IUCD services from *Suraj *centre either 6, 12 and 24 month ago (i.e. in July 2010, December 2009 or January 2009) and were willing to give informed verbal consent were included in the study.

#### Ethical approval

The study protocol was reviewed and approved by the Research & Metrics Department of Marie Stopes International, London, UK.

#### Data collection tool

We adapted and contextually revised a semi-structured questionnaire to capture socio-demographic information, discontinuation rates, reasons for discontinuation, complications and side-effects, follow-up mechanism, accessibility to removal services, service switching behaviour, and level of satisfaction with IUCD and the *Suraj *provider. Similar questionnaire has been used in the studies conducted in Philippines and Vietnam in 2009 and 2010 respectively [17,18]. The same questionnaire was used by MSS in an earlier survey conducted in outreach/rural areas; whereby the tool has already been tested in the similar settings.

#### Data collection and management

The entire data collection was done by a private consultant in January 2011. The local area enumerators were hired and trained on the questionnaire. Face-to-face interviews were conducted at clients' home in privacy. In order to gain more reliable data, before the interview, the local FWM and the enumerator encouraged the client to provide honest responses. On average, each interview took 20 minutes.

Measures were taken to ensure the quality of collected data. All the forms were checked for completeness, logical errors, unclear or irrelevant responses on daily basis. Monitoring visits were also made by the Principal investigator to ensure quality of data and adherence to study protocol.. The data entry and cleaning was done by hiring a different consultant to keep the processes unbiased. The software also restricted for the must filled entries and extreme values. Double entry was performed by two different operators and validated using Visual FoxPro version 6.0.

#### Statistical analysis

Using SPSS 17.0, the descriptive frequencies, proportion and means were run for socio-demographic indicators. Prevalence ratios (PR) were estimated, using Cox regression keeping the time variable constant [19], to see the associations between discontinuation and its risk factors.. All the variables that showed p-value upto 0.25 were included for multivariable analysis [20]. A p-value of less than 0.05 was considered as statistically significant. The data for IUCD insertion and discontinuation data were counted as single-record.

## Case presentation

We approached 3000 women, of those 2789 (92.7%) participated in the study. The reasons for non-response included wrong address, house locked, women migrated or died. There were almost no refusals. The details of stages of sampling are shown in Table 1 below.

**Table 1:** Sampling framework and approach for recruiting women for the study

Multi-stage sampling procedure	1^st ^stage		2^nd ^stage				3^rd ^stage
	
	Randomly selected districts, by stratification of region	→	*Suraj *providers/centres having healthy client flow in July 2010, December 2009 and Jan 2009	→	*Number of clients availed IUCD services in Jul 2010, Dec 2009, and Jan 2009 from the 39 selected providers*	→	Random selection of clients from a list after stratification by type of client
**Selection at each stage**	Nine (9) districts out of 18 districts		Thirty nine (39) *Suraj *providers from **52**		**All **4011 IUCD clients (R-2013; V-1998)		3000 were selected (R-1347; V-1653)

### Socio-demographic characteristics

Of 2789 women interviewed, 76.3% were from Punjab, 67.4% aged > 25 to ≤ 35 years, and had no formal education (59.7%). More than half (56.1%) availed services free of cost (through a voucher). Around 23% women had four children, just ahead of those who had three children (20.2%).

### Information and use of IUCD

Majority of the women had heard about IUCD by word of mouth, mostly from FWM (80.4%), an IUCD client (9%) and from some other sources (3.3%) such as medical camps, posters, family members etc. Similarly, in terms of factors behind choosing IUCD as a long term contraception method, encouragement by FWM or by a satisfied IUCD client is observed to be the most popular reason (43.3%), followed by 'believing in long-term effectiveness' of method (33.1%). Median time to get to the *Suraj *facility was 26 minutes. Moreover, 98% of the IUCD acceptors were informed by providers about the place to refer in case of any complications, and 54.8% did consult a health worker in the community after IUCD insertion. A majority (83.5%) of women opted for IUCD after completing their families, 10.5% opted when they had male child, while only 5.8% opted when they had a female child.

### Experiences of health problems among IUCD acceptors

We found that 634 (22.7%) of the IUCD acceptors experienced some health problem. Of those who faced any health problems, 286 (38.7%) cited heavy bleeding, followed by pain 225 (30.4%) and irregular bleeding 122 (16.5%). Others felt nausea, raised blood pressure, weakness, weight gain etc. Of those who felt some side effect, around half 337 (53.1%) went to *Suraj *centre while 143 (22.5%) did self treatment and 155 (24.4%) reported that they did not require any treatment for these problems.

### Discontinuation of IUCD, reasons, accessibility and level of satisfaction with services

The overall discontinuation rate was reported at 526 (18.0%). We found that 167 (22.7%) women in 24 month cohort, 180 (18.8%) at 12 months cohort and 179 (16.3%) at 6 months cohort discontinued using an IUCD at the time of survey. Of them, the average time of IUCD use was 7.4 (SD ± 5.8). Nearly 22% couldn't recall the duration of use before IUCD removal.

Of the women who discontinued, nearly half 283 (49.8%) of the respondents cited health concerns (including excessive bleeding, pain and irregular bleeding) as the main reason for discontinuation, followed by desire for more children at 193 (34.0%). In addition, 44 (7.7%) discontinued due to their husbands' dislike or in-laws' opposition, whereas 19 (3.3%) switched to permanent method. IUCD expulsion, suggestion by an unhappy IUCD user and method failure were also reported as some of the reasons for IUCD removals with 28 (4.9%).

We found that majority [368 (70.2%)] returned to *Suraj *provider for the removal services, followed by other private clinics at 89 (16.9%). Only 36 (6.8%) got it removed from government facility, 30 (5.7%) reported that it came out itself while the rest sought removal services from lady health worker and traditional birth attendant (*Dai*). Moreover, 513 (97.5%) women found it very easy or not difficult to acquire removal services as it took less than an hour for almost all respondents [478 (90.0%)] to avail removal services. Moreover, 371 (70.5%) got this service free of cost and among those who paid median was PkRs60 (0.69$). We found that, large [2280 (81.7%)] proportion of women expressed willingness to avail IUCD service from *Suraj *provider in future, if needed; whereas 298 (10.7%) women were not willing to use this service in future while the rest were not sure about it. Moreover, 2709 (97.1%) said that they are by and large satisfied and that they will recommend this service to their relatives or friends.

### Factors affecting discontinuation of IUCD

The Univariate analysis in Table 2 shows that women > 35 to ≤ 45 years age group; women not using any contraceptives before IUCD insertion; women experiencing health problem after IUCD insertion; women received IUCD 24 month ago, have high chances of discontinuation. In addition, women living in the proximity of *Suraj *provider and having sons born are more likely to discontinue. We found no significant difference in IUCD discontinuation versus education of the woman or between the women availing the IUCD service free of cost (through voucher) and those paying out of pocket (referred by FWM).

**Table 2:** Association between socio-demographic, services and reproductive factors and IUCD discontinuation

Variables	IUCD Discontinuation			
	
	N	Overall	PR	(95% C.I)
				
		n (%)		
**Region**				

Sindh	689	80 (11.6)	1	--

Southern Punjab	814	194 (23.8)	2.1	1.6-2.7*

Northern Punjab	1286	252 (19.6)	1.7	1.3-2.2*

**Age of women**				

> 15 - ≤ 25	422	72 (17.1)	1	--

> 25 - ≤ 35	1880	344 (18.3)	1.1	0.8-1.4

> 35 - ≤ 45	487	110 (22.6)	1.4	1.01-1.9*

**Number of children**				

≤ 2	590	119 (20.2)	1	--

3	564	93 (16.5)	0.8	0.6-1.1

4	631	104 (16.5)	0.8	0.6-1.1

5 or more	1004	210 (20.9)	1.0	0.8-1.3

**Family status**				

Completed family	5	2 (40.0)	1	--

No children	293	74 (25.3)	2.2	0.6-8.4

Boy(s) only	163	32 (19.6)	1.4	1.1-1.8*

Girl(s) only	2328	418 (18.0)	1.1	0.8-1.6

**Education of women**				

No formal education	1665	298 (17.9)	1	--

Primary	682	133 (19.5)	1.1	0.9-1.3

Secondary	349	74 (21.3)	1.2	0.9-1.5

Inter or post	94	21 (22.3)	1.3	0.8-1.9

**Service availed**				

Referred by FWM (paid out of pocket)	1225	213 (17.4)	1	--

Free of cost (thru voucher)	1564	313 (20.0)	1.2	1.0-1.4

**Type of IUCD**				

Copper-T	790	134 (17.0)	1	--

Multi-load	1999	392 (19.6)	1.2	1.0-1.4

**Reason for choosing IUCD**				

Others	217	31 (14.3)	1	--

Accessibility	277	58 (20.9)	1.4	0.9-2.3

Long term effectiveness side effect	1173	212 (18.1)	1.3	0.9-1.8

Suggested by FWM or satisfied client	1122	225 (20.0)	1.4	1.0-2.0

**Time taken to get to the *Suraj *facility**				

More than 60 minutes	200	16 (8.0)	1	--

30 to60 minutes	683	122 (17.9)	2.5	1.5-4.2*

Less than 30 minutes	1906	388 (20.4)	2.2	1.3-3.8*

**Status of contraception before IUCD insertion**				

Using a contraceptive method	1127	186 (16.5)	1	--

Not using any method	1662	340 (20.5)	1.2	1.04-1.5*

**Experience health problem after IUCD insertion**				

No	2154	236 (11.0)	1	--

Yes	635	290 (45.7)	4.2	3.5-5.0*

Receiving of IUCD				

24 month	736	167 (22.7)	1	--

12 month	957	180 (18.8)	1.1	0.9 -1.5

6 month	1096	179 (16.3)	1.5	1.2 - 1.9*

* Statistically significant

Multivariate analysis in Table 3 shows a high adjusted prevalence ratio of discontinuation for women experiencing any health problem after IUCD insertion; women from both zones of Punjab, women not using any contraception at the time of IUCD insertion and those residing at a distance of less than an hour's travel from the *Suraj *centre.

**Table 3:** Association between socio-demographic, services and reproductive factors and IUCD discontinuation

Variables	APR*	(95% C.I)
**Status of contraception before IUCD insertion**		

Using a contraceptive method	1	--

Not using any method	1.4	1.2-1.7*

**Experience health problem after IUCD insertion**		

No	1	--

Yes	4.5	3.8-5.3*

**Region**		

Sindh	1	--

Southern Punjab	2.0	1.5-2.6*

Northern Punjab	1.7	1.3-2.2*

**Time taken to get to the *Suraj *facility**		

More than 60 minutes	1	--

30 to 60 minutes	2.7	1.7-4.5*

Less than 30 minutes	2.6	1.6-4.4*

Receiving of IUCD		

24 month ago	1	--

12 month ago	1.4	1.1-1.7*

6 month ago	1.2	1.0-1.5

APR: Adjusted prevalence ratio

### Service switching behavior

Of all the IUCD acceptors 1662 (59.6%) were not using any method before the insertion of index IUCD. Out of those who were using some form of contraception before switching IUCD, majority switched from short-term contraceptives such as condoms 455 (16.3%), oral pills 271 (9.7%), injection 250 (9.0%) and withdrawal 129 (4.6%).

Majority of the women who discontinued IUCD [299 (56.8%)], did not switch to any other contraceptives. Others switched to short term method such as condoms 73 (13.9%), pills 34 (6.5%), and injection 15 (2.9%); while 74 (14.1%) and 24 (4.6%) moved to periodic abstinence and tubal ligation, respectively. Among those who didn't switch to another method, nearly half (47%) desired to get pregnant, and31.7% didn't share the reason; while rest of the women cited various reasons such as a forthcoming surgery, husband's demise, fear of side effects etc.

## Discussion

Study shows that IUCD is mainly preferred by women aged between 25 to 35 years, once their desired family size is completed. Physical access seems to be of no issue as most of the women reported travelling less than half an hour to get to the facility for insertion or removal of IUCD, indicating the way program is increasing access to the services for the community. In addition, a significant proportion of the women reported FWM and the satisfied clients as a major source of their knowledge and motivation to use IUCD services which shows the importance of word of mouth, quality of care and the doorstep services by FWMs.

The duration of IUCD use before discontinuation [average 7.4 (SD 5.7) months] among the IUCD acceptors with the main reason being health concerns (including excessive bleeding, pain and irregular bleeding) conforms with the findings of Bangladesh and Vietnam studies [18,21].

The present study demonstrated that age is increasingly associated with discontinuation, while number of children showed no relationship with discontinuation. The findings are similar to the study conducted in Vietnam [18]. However, women education had no association with method discontinuation, which agrees with the Bangladesh study [16]. Moreover, women using a method before IUCD insertion are more likely to continue the method as compared to the women using no method before IUCD insertion. Experiencing any health problem is the most important factor associated with discontinuation. Discontinuation rates among voucher and non-voucher clients were same, which indicates that the vouchers were distributed to eligible women after proper need assessment. Moreover, the higher discontinuation among women may be attributed to the lack of accessibility to removal services as the programme targeted the rural areas, and the dependency of IUCD user on healthcare provider (for insertion and removal). The early discontinuation may be due to side effects, as they normally occur early, following to insertion [22]; therefore it can be expected to see higher early discontinuation followed by low discontinuation. However, in this case 24 month discontinuation was found significantly different from 6 month rate. This could possibly be because of the completion of two years of birth spacing, which is nearly the median birth spacing time in Pakistan [10].

The source of method information and motivation to choose IUCD was the FMW. The discontinuation was low compared to discontinuation rates in other Asian countries [16,18,23,24]. The main reasons for discontinuation included side-effects and desire for more children. The discontinuation rates can be further reduced through training of FWM and *Suraj *provider on counseling techniques. In addition, more frequent follow-up visits should be paid to the clients by FWM during the initial 6-8 month after IUCD insertion to assure and reassure the users that the problems are temporary and are not going to continue for long.

## Conclusion

Such interventions and strong counseling for promoting the use of long term contraceptive methods is one solution to prevent high discontinuation [25]. Such an approach might help in resolving the multi-factoral issue of high unmet need for contraception in Pakistan [26]. In addition, more frequent follow-up visits should be paid to the clients by FWM during the initial 6-8 month after IUCD insertion to assure and reassure the users that the problems are temporary and are not going to continue for long. The study highlights the need to provide counseling to the women to switch to any suitable contraceptive method of their choice, in case they get IUCD removed. Furthermore, a qualitative study should be conducted to appreciate and catalogue the perceptions, beliefs and attitudes of the clients regarding IUCD discontinuation.

## Competing interests

The authors, though, are affiliated with the organization that implemented the programme; however they neither come under nor are not part of programme team. The study was conceptualized and conducted by them independently without any consultation with the implementing team.

## Authors' contributions

SKA & WH was involved in conception and design of the study, analysis and interpretation of the literature, and prepared the draft; BTS added literature and reviewed the analyzed content; MB contributed in revising it critically for substantial intellectual content; GM, WS, IS and AA supervised the data collection, data cleaning and initial analysis. All authors read and approved the final manuscript.

## Pre-publication history

The pre-publication history for this paper can be accessed here:

http://www.biomedcentral.com/1472-6874/12/8/prepub
